# Exploring Sex Differences in Risk Factors and Quality of Life Among Tuberculosis Patients in Herat, Afghanistan: A Case-Control Study

**DOI:** 10.3389/ijph.2024.1606554

**Published:** 2024-04-22

**Authors:** Nasar Ahmad Shayan, Ali Rahimi, Saverio Stranges, Amardeep Thind

**Affiliations:** ^1^ Department of Public Health and Infectious Disease, Faculty of Medicine, Herat University, Herat, Afghanistan; ^2^ Department of Epidemiology and Biostatistics, Schulich School of Medicine and Dentistry, Western University, London, ON, Canada; ^3^ Department of Curative Medicine, Faculty of Medicine, Jami University, Herat, Afghanistan; ^4^ Department of Family Medicine, Schulich School of Medicine and Dentistry, Western University, London, ON, Canada; ^5^ Department of Medicine, Schulich School of Medicine and Dentistry, Western University, London, ON, Canada; ^6^ Department of Clinical Medicine and Surgery, Federico II University, Naples, Italy

**Keywords:** tuberculosis, quality of life, Afghanistan, case-control, Herat, risk factors

## Abstract

**Objectives:** Tuberculosis (TB) is a significant public health concern in Afghanistan, with a high burden of disease in the western province of Herat. This study explored the risk factors of TB and TB’s impact on the quality of life of patients in Herat.

**Methods:** A total of 422 TB patients and 514 controls were recruited at Herat Regional Hospital and relevant TB laboratories between October 2020 and February 2021. Data was collected through interviews using a structured questionnaire and the SF-36 questionnaire. Descriptive statistics, chi-square tests, Multivariate General Linear Model, and logistic regression analysis were used to analyze the data.

**Results:** The results showed that male sex (*p* = 0.023), chronic disease (*p* = 0.038), lower education levels (*p* < 0.001), and worse health status (*p* < 0.001) were significantly associated with higher odds of TB infection. The study also found that TB patients had significantly lower quality of life scores in almost all components (*p* < 0.05).

**Conclusion:** This study provides important insights into the specific ways in which TB affects the wellbeing of patients in Afghanistan. The findings highlight the importance of addressing the psychological and social dimensions of TB.

## Introduction

Tuberculosis (TB) is a significant public health concern worldwide, with an estimated 10.6 million cases and 1.6 million deaths in 2021 according to the World Health Organization (WHO) [1]. The disease is caused by Mycobacterium *tuberculosis* (MTB) and is transmitted through droplets in the air. Individuals with poor immune defenses, such as those infected with Human Immunodeficiency Virus (HIV), diabetics, and those receiving immunosuppressive therapy, are at a higher risk of developing the disease [2]. Despite efforts to improve case identification and treatment compliance, the incidence of TB remains high in many low-income countries, with the WHO aiming to decrease the incidence rate by 4%–5% per year [1].

In Afghanistan, TB is a particularly pressing issue, particularly in the western province of Herat [3, 4]. In 2016, an estimated 65,000 cases and 11,000 deaths were caused by TB in Afghanistan, with 47,406 cases detected and enrolled in treatment in 2017 [5]. Out of all health facilities (*n* = 2,857), 71% are providing Directly Observed Treatment (DOTS) services [5]. Despite efforts to improve case identification and treatment compliance, TB remains a leading cause of death from infectious diseases in Afghanistan and worldwide, second only to HIV [6].

The impact of TB on the quality of life of patients is a complex and multifaceted issue, with physical, psychological, and social dimensions [7, 8]. The physical symptoms of TB, such as coughing, fever, and weight loss, can lead to significant declines in physical functioning and wellbeing [9]. Additionally, the psychological and social consequences of TB, such as stigma, discrimination, and isolation, can further negatively impact the quality of life of patients [10]. Furthermore, TB patients often experience high levels of anxiety and depression related to their diagnosis and treatment, as well as the potential impact of the disease on their ability to work, care for their families, and maintain social relationships [11, 12]. These psychological symptoms can have a significant impact on the overall wellbeing of patients, and can further exacerbate physical symptoms, leading to a vicious cycle of poor health and reduced quality of life [7].

Despite the high burden of TB in Afghanistan and the potential negative impact on the quality of life of patients, there is limited research on the topic in the region [13, 14]. This study aims to fill this research gap by exploring potential risk factors of TB and assessing its profound impact on the quality of life of patients in Herat, Afghanistan. The unique context of Afghanistan as a developing country, particularly in Herat, further emphasizes the significance of this research endeavor.

In the context of Afghanistan’s development, understanding the specific challenges posed by TB in Herat is crucial for informed public health interventions. Factors such as socioeconomic conditions, healthcare infrastructure, and cultural dynamics play a pivotal role in shaping the experiences of TB patients. By delving into these factors through a case-control study design, this research not only contributes to the global understanding of TB but also provides context-specific insights that can inform targeted strategies for prevention, treatment, and support in Herat. Consequently, the outcomes of this study have the potential to guide tailored interventions that address the unique needs of TB patients in this specific region, ultimately contributing to the improvement of public health outcomes in Afghanistan.

### Literature Review

A growing body of research has explored risk factors and quality of life impact associated with TB in different global settings. A systematic review of 25 observational studies found that illiteracy, low income, indoor air pollution, smoking, diabetes, and HIV infection are significant risk factors for TB across multiple countries [15]. The review highlighted socioeconomic disparities as key drivers of TB susceptibility worldwide.

Several studies have also examined the effects of TB on patients’ quality of life using validated assessment tools. A cross-sectional study in Bangladesh applied the SF-36 questionnaire to compare quality of life between TB patients and healthy controls [2]. TB patients reported significantly lower overall quality of life, which improved by treatment. A Canadian cohort study using the SF-36 revealed that TB patients scored lower on all quality of life subscales, particularly in the weeks following diagnosis and treatment initiation, with improvement observed after 2 months [16].

While Afghanistan suffers from a high TB burden, few studies have explored associated factors and patient-reported outcomes in this setting. A hospital review of extrapulmonary TB cases in Kabul found a majority were women, indicating potential gender differences [13]. Studies in Kabul revealed knowledge gaps and stigmatizing attitudes about TB among Afghans in Pakistan [17, 18].

In summary, the literature demonstrates TB’s multidimensional impact on quality of life across diverse geographic and cultural contexts. However, data specific to Afghanistan is scarce. This study addresses this gap by examining TB risk factors and quality of life among patients in Herat. The findings can guide targeted interventions to improve prevention and care for this vulnerable population.

## Methods

### Study Population

A case-control study was conducted to explore the impact of TB on the quality of life of patients in Herat, Afghanistan. The study was approved by the research committee on TB research at Herat Regional Hospital and three related TB laboratories. The study was conducted between October 2020 and February 2021 at the TB department of Herat Regional Hospital in Herat city. Over 90% of patients visited Herat Regional Hospital directly, while the remaining 10% were referred from three laboratories but retested at Herat Regional Hospital. The study population consisted of bacteriologically confirmed pulmonary TB patients with ages above 18 years who were fluent in Dari (the Persian dialect used in Afghanistan). The cases were newly registered TB patients who were diagnosed in the laboratory. A total of 422 patients were recruited for the study. The control group consisted of age -matched individuals without a history of TB who were also fluent in Dari. The control group was selected from patients who presented to the Herat Regional Hospital as the cases for non-TB health problems (internal medicine and non-infectious disease). A total of 514 controls were recruited for the study.

### Sampling Procedure

• **Selection of cases:** The cases were newly detected bacteriologically confirmed pulmonary TB patients aged above 18 years, enrolled for treatment in the selected clinics in Herat city. Data collection was conducted by selecting newly registered TB patients using convenience sampling, based on the availability and accessibility of participants within a specified timeframe and location.
• **Selection of controls:** Age-matched individuals without a history of TB were selected as controls from the Herat Regional Hospital and three relevant laboratories. The participants were approached and asked to participate in the study if they met the age and sex criteria. If an individual refused to participate, another person who met the criteria was approached using convenience sampling within the specified timeframe and location. The goal was to ensure a larger number of participants in the case group compared to the control group.

### Data Collection

Data was collected using a structured questionnaire that consisted of three sections, including individual-socio-demographic characteristics (26 items), signs, symptoms, and effects of TB (36 items), and the SF-36 questionnaire (36 items).

• **Socio-demographic section (26 items)**: The individual-economic-social characteristics section of the questionnaire included information on ID, name, father’s name, case or control status, age, weight (kg), height (cm), gender, marital status, education, occupation status, economic status, self-reported health status, nutrition status, smoking, presence of chronic disease, and type of chronic disease.
• **Signs, symptoms, and effects of TB (36 items):** This section included questions regarding the presence or absence of TB, any testing for TB, and the timing and duration of symptoms (cough, chest pain, fever, appetite, nightly sweating, expectoration, hemoptysis, weight loss). The authors developed a signs and symptoms questionnaire through a review of TB studies and collaboration with infectious disease professionals [19, 20]. A pilot study, involving 30 TB patients, was conducted to evaluate its performance.
• **SF-36 section (36 items):** The SF-36 questionnaire is a widely used tool for measuring health-related quality of life [21], and has been validated in many languages and cultures, including the Dari language in Herat, Afghanistan [22]. In this validation study, the SF-36 questionnaire demonstrated good construct validity, internal consistency, and test-retest reliability, with Cronbach’s alpha values ranging from 0.753 to 0.933 for the 8 subscales. The eight dimensions encompass physical functioning (PF), role limitations due to physical health problems (RLPH), bodily pain (BP), social functioning (SF), role limitations due to emotional problems (RLEP), general mental health (MH), vitality (VT), and general health perceptions (GH). The questionnaire evaluates both physical and mental aspects, with the Physical Component Subscale (PCS) covering PF, GH, BP, and RLPH, and the Mental Component Subscale (MCS) covering MH, SF, VT, and RLEP. The questionnaire was administered in person by trained interviewers and the data was collected confidentially.

### Data Analysis

Data were analyzed using descriptive and inferential statistics. The chi-square test was used to compare the differences in categorical variables between the case and control groups. Furthermore, significant factors identified through the chi-square test, associated with the development of TB, were then entered into a logistic regression model analysis to ascertain their odds ratios for TB development. The Multivariate General Linear Model was used to compare the differences in the mean quality of life scores between TB patients and the control group, separately for men and women, while controlling for the impact of other factors on quality of life. The statistical significance limit (*p*-value) for all tests was set at 0.05. Additionally, the normal distribution of continuous variables was assessed in accordance with established methods. The data were analyzed using the statistical software package SPSS version 26.

### Ethical Considerations

The study was approved by the Human Ethics Committee, Bureau of Research and Development, Faculty of Medicine, Herat University on 20 January 2020. All participants provided written informed consent before participating in the study, including those who were illiterate, where their respective legally authorized representatives (LARs) were involved in the informed consent process. The confidentiality and privacy of the participants were protected throughout the study, following the Declaration of Helsinki and the ethical principles of research involving human subjects.

## Results

The socio-demographic characteristics of 936 participants, including 422 TB patients and 514 controls are presented in Table 1. The mean (SD) and the median age of the TB patients were 31.0 (±15) and 35.6 years respectively, while for the control group, it was 29.0 (±13.7) and 33.3 years respectively.

**TABLE 1:** General characteristics of tuberculosis patients and controls (Herat, Afghanistan, 2021).

Characteristics	Case		Control		*p*
	N	%	N	%	
Sex					
Female	212	50.2	297	57.8	**0.014**
Male	210	49.8	217	42.2	
Age group					
18–29	183	43.4	263	51.2	0.212
30–39	101	23.9	110	21.4	
40–49	55	13.0	58	11.3	
50–59	39	9.2	39	7.6	
60 and above	44	10.4	44	8.6	
Marital Status					
Currently not married	91	21.6	243	47.3	**<0.001**
Currently married	331	78.4	271	52.7	
Education					
Illiterate	178	42.2	68	13.2	**<0.001**
Just read and write	80	19.0	43	8.4	
Primary	42	10.0	32	6.2	
Secondary	39	9.2	32	6.2	
Tertiary	56	13.3	88	17.1	
University	27	6.4	251	48.8	
Occupational status					
Employed	146	34.6	193	37.5	0.350
Unemployed	176	65.4	321	62.5	
Economic Status					
Very good	7	1.7	39	7.6	**<0.001**
Good	69	16.4	199	38.7	
Average	177	41.9	230	44.7	
Bad	129	30.6	37	7.2	
Very bad	40	9.5	9	1.8	
Health Status					
Very good	8	1.9	128	24.9	**<0.001**
Good	67	15.9	263	51.2	
Average	129	30.6	100	19.5	
Bad	190	45.0	20	3.9	
Very bad	28	6.6	3	0.6	
Nutrition Status					
Very good	10	2.4	96	18.7	**<0.001**
Good	86	20.4	242	47.1	
Average	178	42.2	159	30.9	
Bad	122	28.9	16	3.1	
Very bad	26	6.2	1	0.2	
Chronic disease					
Yes	147	34.8	68	13.2	**<0.001**
No	275	65.2	446	86.8	
BMI					
Underweight	75	17.8	45	8.8	**<0.001**
Normal	252	59.7	318	61.9	
Overweight	80	19.0	123	23.9	
Obesity	15	3.6	28	5.4	
Cigarette smoking					
Yes	112	26.5	73	14.2	**<0.001**
No	310	73.5	441	85.8	
Total	**422**	**100.0**	**514**	**100.0**	

*p<0.05, that indicate that the corresponding results or comparisons are statistically significant.

The study found that a majority of the participants in both the case and control groups were female, with 50.2% and 57.8% respectively. The difference in sex between the two groups was statistically significant (*p* = 0.014). A larger proportion of the case group was married (78.4%), while a significant number of the control group were not married (47.3%) (*p* < 0.001). Similarly, the majority of the control group had at least a tertiary level education (48.8%), while most participants in the case group were illiterate (42.2%) (*p* < 0.001). A larger proportion of the control group had a “good” or “very good” economic status (46.3%) compared to the case group (18.1%) (*p* < 0.001). Similarly, a larger proportion of the case group reported a “bad” or “very bad” health status (51.6%) compared to the control group (4.5%) (*p* < 0.001). The study also found a significant difference in the nutrition status between the two groups, with a larger proportion of the control group having a “good” or “very good” nutrition status (65.8%) compared to the case group (22.8%) (*p* < 0.001). Additionally, a larger proportion of the case group reported having a chronic disease (34.8%) compared to the control group (13.2%) (*p* < 0.001). The study found a significant difference in Body Mass Index (BMI) distribution between the two groups, with a larger proportion of the control group having a normal BMI (61.9%) compared to the case group (59.7%) (*p* < 0.001). Lastly, a larger proportion of the case group reported being smokers (26.5%) compared to the control group (14.2%) (*p* < 0.001). No differences were noted between the groups in age distribution and occupational status (Table 1).

Males and females had similar signs and symptoms (Table 2). The only statistically significant differences were between night sweats (*p* = 0.014) and weight loss (*p* = 0.032), where women were more likely to report these.

**TABLE 2:** Frequency and percentage of signs and symptoms of tuberculosis cases by sex (Herat, Afghanistan, 2021).

Sign and symptom	Male		Female		*p*
	N	%	N	%	
Cough					
Yes	173	82.4	185	87.3	0.162
No	37	17.6	27	12.7	
Fever					
Yes	136	64.8	151	71.2	0.155
No	74	35.2	61	28.8	
Loss of appetite					
Yes	132	62.9	141	66.5	0.433
No	78	37.1	71	33.5	
Night sweats					
Yes	109	51.9	135	63.7	**0.014**
No	101	48.1	77	36.3	
Weight loss					
Yes	99	47.1	122	57.5	**0.032**
No	111	52.9	90	42.5	
Expectoration					
Yes	89	42.4	103	48.6	0.201
No	121	57.6	109	51.4	
Chest Pain					
Yes	85	40.5	102	48.1	0.114
No	125	59.5	110	51.9	
Hemoptysis					
Yes	33	15.7	35	16.5	0.824
No	177	84.3	177	83.5	
Total	**210**	**100.0**	**212**	**100.0**	

*p<0.05, that indicate that the corresponding results or comparisons are statistically significant.

This study utilized logistic regression analysis to investigate significant variables associated with TB infection, with the dependent variable being the TB status (case or control). The significant variables were selected from Table 1 and included sex, chronic disease, smoking status, marital status, education level, health status, nutrition status, economic status, and BMI status. The odds ratio (OR) with a 95% confidence interval (CI) was calculated for each significant variable. The results showed that males had higher odds of TB infection than females (OR = 1.60, 95% CI = 1.07–2.41, *p* = 0.023); participants with chronic disease also had higher odds than those without (OR = 1.62, 95% CI = 1.03–2.54, *p* = 0.038). Illiterate participants had the highest odds of infection (OR = 8.31, 95% CI = 4.34–15.89, *p* < 0.001), followed by those who could only read and write (OR = 8.79, 95% CI = 4.41–17.54, *p* < 0.001), those in primary school (OR = 4.81, 95% CI = 2.27–10.17, *p* < 0.001), those in secondary school (OR = 6.30, 95% CI = 3.05–13.02, *p* < 0.001), and those in tertiary school (OR = 3.32, 95% CI = 1.76–6.23, *p* < 0.001). Participants in worse health status were found to have higher odds of TB infection, with those in average health status having the highest odds of infection (OR = 7.91, 95% CI = 3.08–20.35, *p* < 0.001), followed by those in bad health status (OR = 34.23, 95% CI = 12.05–97.20, *p* < 0.001), and those in very bad health status (OR = 27.07, 95% CI = 14.94–148.16, *p* < 0.001).

No significant differences were found for cigarette smoking, marital status, nutrition status, BMI status, or economic status (Table 3).

**TABLE 3:** Logistic regression model assessing tuberculosis risk in cases compared to controls (Herat, Afghanistan, 2021).

	*p*-value	OR	95% C.I. for OR	
			Lower	Upper
Sex				
Male	**0.023**	1.603	1.066	2.409
(Ref) Female				
Chronic Disease				
Yes	**0.038**	1.616	1.026	2.544
(Ref) No				
Cigarette				
Yes	0.727	1.090	0.673	1.766
(Ref) No				
Marital Status				
Single	0.959	1.012	0.637	1.609
(Ref) Married				
Education				
Illiterate	**<0.001**	8.313	4.347	15.896
Just read and write	**<0.001**	8.793	4.407	17.544
Primary	**<0.001**	4.810	2.274	10.174
Secondary	**<0.001**	6.302	3.051	13.016
Tertiary	**<0.001**	3.315	1.762	6.236
University (Ref)				
Health Status				
(Ref) Very good				
Good	0.125	2.066	0.819	5.214
Average	**<0.001**	7.913	3.077	20.351
Bad	**<0.001**	34.226	12.052	97.202
Very bad	**<0.001**	27.067	4.945	148.165
Nutrition Status				
(Ref) Very good				
Good	0.650	1.249	0.477	3.270
Average	0.588	1.314	0.490	3.523
Bad	0.058	3.061	0.964	9.720
Very bad	0.073	9.730	0.812	116.586
Economic Status				
(Ref) Very good				
Good	0.888	0.912	0.253	3.283
Average	0.711	0.785	0.218	2.827
Bad	0.644	1.377	0.355	5.342
Very bad	0.636	0.673	0.131	3.469
BMI (Normal)				
Underweight	0.327	1.344	0.744	2.428
(Ref) Normal				
Overweight	0.093	0.674	0.426	1.067
Obese	0.120	0.494	0.203	1.203

Enter Method, Hosmer and Lemeshow test = 0.238, Cox and Snell R Square = 0.443, Nagelkerke R Square = 0.593, Omnibus test of Model Coefficients = 0.000, OR: Odds Ratio. *p<0.05, that indicate that the corresponding results or comparisons are statistically significant.

Table 4 displays the mean scores of SF-36 components for TB cases and controls, adjusted for demographic and health factors, and stratified by sex. The results showed that, for males, TB cases had significantly lower mean scores in all SF-36 components except for mental health, with highly significant differences (*p* < 0.001). Similarly, for females, TB cases had significantly lower mean scores in all SF-36 components, with significant differences (*p* < 0.05). TB status was associated with lower scores in various aspects of quality of life, with the largest differences observed in role limitations due to physical health and role limitations due to emotional problems for both males and females. Furthermore, the differences in the quality of life between cases and controls were more prominent among males than females. The difference in mean scores for quality of life between female cases and controls across all SF-36 components was generally smaller compared to males, except for the domain of role limitation due to emotional problems.

**TABLE 4:** Mean scores of the Short Form-36 component Tuberculosis case and control by sex (Herat, Afghanistan, 2021).

Sex/TB status	Fully adjusted				p*
	Case		Control		
	Mean	Standard. Error	Mean	Standard. Error	
**Male**	**Case (297)**		**Control (210)**		
SF-36 (Quality of Life)				
Physical functioning	60.49	1.95	75.31	1.54	**<0.001**
Role limitation due to physical health	43.80	2.65	73.44	2.09	**<0.001**
General Health	50.32	1.85	68.07	1.46	**<0.001**
Bodily pain	66.14	1.67	79.80	1.31	**<0.001**
Mental health	69.68	1.89	68.80	1.49	0.749
Vitality	48.31	2.05	66.29	1.61	**<0.001**
Role limitation due to emotional problems	36.08	3.12	69.80	2.45	**<0.001**
Social functioning	61.56	1.79	76.08	1.41	**<0.001**
PCS (Physical component summary)	55.19	1.42	74.15	1.12	**<0.001**
MCS (Mental component summary)	53.91	1.56	70.24	1.23	**<0.001**
**Female**	**Case (217)**		**Control (212)**		
SF-36 (Quality of Life)				
Physical functioning	54.40	1.84	70.46	1.75	**<0.001**
Role limitation due to physical health	33.13	2.62	67.01	2.50	**<0.001**
General Health	43.39	1.76	59.47	1.69	**<0.001**
Bodily pain	57.67	1.75	76.87	1.67	**<0.001**
Mental health	59.48	2.11	67.97	2.01	**0.011**
Vitality	39.40	2.01	57.25	1.92	**<0.001**
Role limitation due to emotional problems	27.88	2.98	63.67	2.84	**<0.001**
Social functioning	53.75	1.79	74.78	1.71	**<0.001**
PCS (Physical component summary)	47.15	1.35	68.45	1.29	**<0.001**
MCS (Mental component summary)	45.13	1.56	65.92	1.49	**<0.001**

Adjusted for Age, Marital status, Education, Economic status, Health status, Nutrition status, and chronic diseases. *p<0.05, that indicate that the corresponding results or comparisons are statistically significant.

## Discussion

This case-control study on the risk factors and quality of life among TB patients in Herat, Afghanistan represents a significant contribution to the scientific literature on this topic. To our knowledge, this is the first study of its kind to be conducted in Afghanistan, making our findings especially noteworthy. Our regression model revealed several factors that exhibited significant associations with the disease, and we will delve into these in the following section.

### Factors that showed significant associations in the regression model

The regression model showed that males had 60% greater odds of getting TB than females, which is consistent with other studies reporting higher notification rates for men than women [23]. This is consistent with other studies reporting higher notification rates for men than women: 60.50% [24], 57.50% [25], 70.70% [26], and 71.10% [27]. However, a study in Kabul reported a 2:1 female-to-male ratio, while a similar finding with a more prominent difference was observed in another study where (43.30%) of participants were males [28]. According to a study by WHO, men are more likely to be diagnosed with TB than women, with a global ratio of 1.6:1 [29], although this ratio varies by region and age group. The sexual dimensions of TB also vary by age group. For example, one study found that among young adults (15–34 years), men had a higher risk of TB than women, but among older adults (65+ years), women had a higher risk than men [30]. It is important to examine sex factor when studying TB epidemiology and interventions and dedication of resources.

The higher TB burden in males may be due to biological or social differences, such as exposure to TB infection, access to healthcare, or diagnostic practices [29]. Apart from biological factors, socio-economic and cultural factors also contribute to sex differences in TB [31, 32]. Poverty, stigma, discrimination, violence, and access to resources are social determinants of TB that need to be addressed [33]. Women face additional challenges in TB control, such as being diagnosed at a later stage of the disease, having lower treatment completion rates, and facing more social stigma than men [34]. Therefore, sex-based approaches should be integrated into TB control programs to address these challenges and reduce the burden of TB. Further research is needed to better understand the reasons for the higher TB burden in males and to inform gender-sensitive interventions for TB control. It is worth noting that since the Taliban took power in Afghanistan women have been banned from work and education, which may further exacerbate discrimination against women in accessing health and TB resources.

The findings of this study showed that individuals with lower education levels had greater odds of TB infection. The risk of TB infection decreased gradually with higher education levels. This may be because higher education leads to a higher level of understanding of health and better attitudes toward disease prevention efforts [35]. Previous studies have shown that individuals with low knowledge about TB transmission and prevention had a greater risk of being infected with TB compared to those with high knowledge [36]. Education could be a risk factor for developing TB for several reasons. Lower levels of education could lead to low awareness about the disease and its prevention. Furthermore, people who are in close contact with someone who has infectious TB may not have adequate knowledge or resources to protect themselves or seek treatment [37]. According to some studies, different levels of education may have different effects on TB risk. For example, one study found that higher education was associated with lower TB incidence and mortality in low- and middle-income countries [38]. Another study found that lower education was associated with higher TB prevalence [39]. However, these associations may vary depending on other factors such as socioeconomic status, health system performance, and cultural norms. Overall, improving education and awareness about TB could help reduce its burden and improve its control globally.

Our study found that having underlying chronic diseases such as COPD, diabetes, heart failure, kidney disease, and liver disease is significantly associated with greater odds of having TB. This was supported by the regression model that showed people with underlying chronic disease had significantly lelo1.616 times greater odds of contracting TB. This result is consistent with previous studies that have demonstrated the link between chronic diseases and the risk of TB [40–42]. People with chronic diseases are at a higher risk of contracting TB due to weakened immune systems that make them more susceptible to infections. Chronic diseases can also cause inflammation and damage to the lungs, which creates an ideal environment for TB bacteria to thrive [43].

Our study also found a significant association between perceived health status and TB infection. Participants who reported being in worse health status were more likely to have TB infection than those who reported being in better health. Perceived health status is a complex construct that can be influenced by various factors, including physical, psychological, and social aspects. Previous research has shown that individuals who perceive their health to be poor are more likely to engage in unhealthy behaviors and have poorer health outcomes than those who perceive their health to be good [44, 45]. While we were not able to rule out reverse causality, interventions aimed at improving individuals’ health status may also have positive effects on reducing TB risk and improving TB outcomes.

### Health-Related Quality of Life

This study found highly significant differences between TB cases and controls in each subdomain of SF-36 quality of life. TB has a remarkable negative impact on several dimensions of health-related quality of life (HRQoL) for TB patients, as seen in other studies [16, 46–48]. The SF-36 scale scores for patients in this study ranged from 30.10 to 62.53, with the overall physical and mental health scores being 48.34 and 47.47, respectively. They were noticeably higher than studies in South Africa (ranging from 42.5 to 40.7) [49] and Iran (ranging from 14.68 to 46.99) [50], which may be due to differences in environmental study and pathology of TB disease.

The HRQoL scores of patients were analyzed in different dimensions and it was found that patients scored low in the role limitation-physical (36.47) and role limitation-emotional (30.10) dimensions compared to scores from previously published studies. For example, the scores were 65 in Iran and 60.0 in Pakistan for role limitation-physical and 45 in Iran, and 59.01 in Pakistan for role limitation-emotional [47, 51]. This indicates that TB can lead to depression and anxiety, which can contribute to a decline in patients’ overall health status. A study by Laxmeshwar et al. [52] and others have revealed that both the physical and psychological health domains of TB patients are significantly affected by the disease. Additionally, studies by Mamani et al. [47], Unalan et al. [53], and Duyan et al. [54] have shown that depression is more common among TB patients than in non-TB groups, and this is highly associated with low HRQoL scores [55].

Even though most patients reported low HRQoL scores, the underlying reason for this could be attributed to the psychological impacts of the disease, such as social isolation and rejection by the community and family due to the contagious nature of the disease [56, 57]. These findings also highlight the negative impact of TB on quality of life and suggest that interventions to address the physical and emotional effects of the disease are necessary to improve the wellbeing of TB patients.

### Policy and Implications

The insights from our study hold significant policy implications for TB prevention and control programs, especially considering the prevailing ground realities and the influence of the new government with its orthodox policies. In this context, it becomes imperative to craft policies that are not only effective but also realistic, considering the current political landscape.

Identifying and treating chronic diseases in individuals at high risk of TB gains heightened importance, particularly in countries grappling with a substantial TB burden. The integration of TB screening into the management of chronic diseases is a practical approach that aligns with the new government’s policies. Additionally, emphasizing the importance of TB prevention measures, such as infection control and early detection, among patients with chronic diseases is crucial.

Given the orthodox policies of the new government, it is essential to align TB control programs with their priorities. A strategic focus on identifying and treating individuals with chronic diseases, particularly those at a heightened risk of TB, can serve as a realistic and impactful approach. By doing so, TB control programs can contribute significantly to reducing the overall burden of TB in the population, aligning with the current governmental approach and fostering improved public health outcomes [58].

### Limitations

Although this study provides valuable insights into the TB-associated factors and the impact of TB on the quality of life of patients in Herat, Afghanistan, some limitations should be acknowledged. Firstly, the study’s focus on a single region and solely pulmonary TB patients, the most common form of TB worldwide, may limit the generalizability of the findings to other regions or types of TB. Additionally, the study did not assess the impact of comorbidities on the quality of life of TB patients, which could have provided additional valuable information. Given the observational, case-control design, the study suffers from several intrinsic limitations, including residual confounding by unmeasured or unknown factors, which may have affected tuberculosis risk and quality of life in our study population. Despite these limitations, the study offers important insights into the impact of TB on patients’ quality of life in Herat, Afghanistan, emphasizing the need for further research in this area.

### Conclusion

This case-control study examines the role of potential risk factors for tuberculosis as well as the quality of life of TB patients in Afghanistan. The study provides important insights that can inform national policies and healthcare resource allocation to address TB transmission and improve patient management. Results reveal that Illiteracy and low economic status are significant risk factors for TB infection, with education level identified as the most significant predictor of infection. Patients with lower economic status also have a higher risk of infection. These findings emphasize the need for targeted intervention programs for vulnerable groups, and future research is needed to investigate the link between marital status and TB infection. Additionally, this study revealed significant disparities in Health-Related Quality of Life between TB cases and controls, with TB patients experiencing notable negative impacts on various dimensions, suggesting a need for interventions addressing both the physical and psychological effects of TB to enhance patient wellbeing. Ultimately, the study provides evidence to inform evidence-based healthcare policies and practices that can improve the quality of life for TB patients in Afghanistan and other countries facing high TB burdens.

## Data Availability

The datasets used and/or analyzed during the current study are available from the corresponding author upon reasonable request.

## Abbreviations

TB, Tuberculosis; MTB, Mycobacterium *tuberculosis*; HRQoL, health-related quality of life; HIV, Human Immunodeficiency Virus; WHO, World Health Organization; COPD, Chronic Obstructive Pulmonary Disease.
